# PAU/RAD: Design and Preliminary Calibration Results of a New L-Band Pseudo-Correlation Radiometer Concept

**DOI:** 10.3390/s8074392

**Published:** 2008-07-28

**Authors:** Xavier Bosch-Lluis, Adriano Camps, Isaac Ramos-Perez, Juan Fernando Marchan-Hernandez, Nereida Rodriguez-Alvarez, Enric Valencia

**Affiliations:** Remote Sensing Lab, Dept. Teoria del Senyal i Comunicació, Campus Nord D3, Universitat Politècnica de Catalunya, 08034 Barcelona, Spain

**Keywords:** radiometer, reflectometer, pseudo-correlation, calibration, field-programmable gate array (FPGA)

## Abstract

The Passive Advanced Unit (PAU) for ocean monitoring is a new type of instrument that combines in a single receiver and without time multiplexing, a polarimetric pseudo-correlation microwave radiometer at L-band (PAU-RAD) and a GPS reflectometer (PAU-GNSS/R). These instruments in conjunction with an infra-red radiometer (PAU-IR) will respectively provide the sea surface temperature and the sea state information needed to accurately retrieve the sea surface salinity from the radiometric measurements. PAU will consist of an array of 4×4 receivers performing digital beamforming and polarization synthesis both for PAU-RAD and PAU-GNSS/R. A concept demonstrator of the PAU instrument with only one receiver has been implemented (PAU-One Receiver or PAU-OR). PAU-OR has been used to test and tune the calibration algorithms that will be applied to PAU. This work describes in detail PAU-OR's radiometer calibration algorithms and their performance.

## 1. Introduction

Sea surface salinity can be indirectly measured through the variations of the brightness temperature due to the change of the sea water dielectric constant with respect to temperature and salinity [1]. However, the brightness temperature also depends on the sea surface roughness. Despite the field experiments performed in the past years to improve our understanding of this effect [2,3,4], the sea surface roughness correction still remains one of the most critical corrections needed to retrieve the salinity with the required accuracy since it cannot be simply parameterized in terms of the wind speed and/or the significant wave height. Inaccuracies in the brightness temperature direct model may induce significant errors (biases) in the retrieved salinity [5].

**Figure 1.**
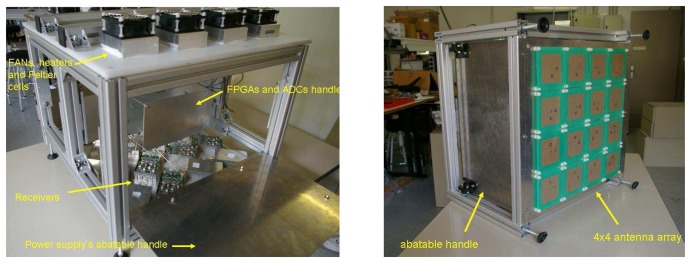
PAU overview.

The Passive Advanced Unit (PAU) (Fig. 1) is a new concept instrument originally proposed in 2003 [6]. PAU consists of a suite of three instruments operating synergistically:

- PAU-RAD: a new type of pseudo-correlation L-band radiometer to measure the brightness temperature of the sea surface,
- PAU-GNSS-R: a GPS-reflectometer to measure the sea state, and
- PAU-IR: an IR radiometer to measure the sea surface temperature.

PAU-RAD is a full polarimetric radiometer, so that the delivered results are the four Stokes parameters (eqn. 1). The Stokes parameters fully characterize the polarization state of partially polarized thermal radiation. This polarized thermal radiation is related to different phenomena such as the angle incidence, the sea surface salinity (SSS), the sea surface temperature (SST), the sea surface roughness (SSR), wind direction, foam…

(1) $$\bar{I}=\left[\begin{array}{c} I\\ Q\\ U\\ V \end{array}\right]=\left[\begin{array}{c} {\left|\;E_{v}\;\right|}^{2}+\;{\left|\;E_{h}\;\right|}^{2}\\ {\left|\;E_{v}\;\right|}^{2}-\;{\;|\;E_{h}\;|}^{2}\\ 2\cdot ℜe<E_{v}\cdot E_{h}^{*}>\\ 2\cdot ℑm<E_{v}\cdot E_{h}^{*}> \end{array}\right]\;,$$

where:

- *I*,*Q*,*U* and *V* are the four Stokes parameters, and
- *E_v_* and *E_h_* are the electric fields at vertical and horizontal polarizations.

The first and the second parameters (*I*, *Q*) are the sum and the difference of the brightness temperatures at vertical and horizontal polarizations. It is known that the sea surface salinity can be retrieved from *I* and *Q* [1]. The third and the fourth parameters (*U*, *V*) characterize the correlation between these two orthogonal polarizations. Wind direction information can be retrieved from *U* and *V* [7].

The GPS-reflectometer uses the same RF-IF front-end as the L-band radiometer, although the digital beamformer synthesizes beams pointing towards the specular reflection points of the GPS signals [8,9]. The relationship of the standard deviation of the SSR and the brightness temperature is obtained $\left(\frac{\Delta T_{B}}{\sigma_{\text{SSR}}}\right)$ [10] from this instrument. Finally, the third element is a commercial 8-14 mm thermal IR radiometer [11], from which the SST is obtained. PAU [12] will retrieve the SSS from radiometer outputs (Stokes parameters) and correcting the sea state effect using information from the reflectometer $\left(\frac{\Delta T_{B}}{\sigma_{\text{SSR}}}\right)$ and from the IR-radiometer (SST).

PAU-RAD consists of an array of 4×4 dual polarization receivers integrated behind a patch antenna [13], whose outputs are digitized, and then properly calibrated and combined to produce several beams using a digital beamformer [14]. In this implementation the PAU-RAD operates at the GPS L1 frequency, sharing the RF-IF front-end with the PAU-GNSS/R. PAU-RAD's digital design has been split in two Field Programmable Gate Array (FPGA) [14]: one is called Control Unit (CU) and manages the system and the another one is called Arithmetic-Logic Unit (ALU) and implements the radiometer algorithms.

Section 2 presents the receiver design and operation. Section 3 describes the calibration algorithms implemented, which includes the injection of correlated noise at two different levels, and a Kalman filter [15] to better track the phase drifts. Calibration results are presented at the end of this section. Finally, section 4 presents the main conclusions and future research lines.

## 2. PAU-RAD design overview

Each element in the array has a dual-polarization antenna (horizontal and vertical) and each polarization is thereafter divided in two using a Wilkinson power splitter. The PAU radiometer idea is that the output voltage signals are proportional to the antenna signal plus or minus the noise added by the resistor of the Wilkinson power splitter. A detailed noise wave analysis of this novel topology is described in [12]. The receiver topology is described in detail in [13]: The input signals are demodulated at an intermediate frequency (IF) of 4.309 MHz and have a 2.2 MHz bandwidth. The analog signals are then digitalized at 8 bits at a sampling frequency of 5.745 MHz.

To avoid the mechanical scan of the antenna, a digital beamformer with the 4×4 element array has been designed so that the beam can be steered up to ±20° from the array boresight (45° incidence angle) in 5° steps (incidence angle from 25° to 65°). The PAU-RAD basic requirements are summarizes below:

- I/Q demodulation of 64 input signals (16 elements × 2 polarization/element × 2 receiving chains/polarization),
- maximum amplitude unbalance within ±1 dB. Residual amplitude unbalance better than ±0.1 dB. Limited by the number of bits,
- phase calibration between receiving chains better than 2° rms (random initial error within 0° and 360°),
- computation the four Stokes parameters using correlation techniques,
- system re-configurability, so that the integration and calibration times, and the inter-calibration period may be optimized as a function of the scenario and application, and
- simultaneous generation of two simultaneous beams, with the computing resources available.

As it has been said in the introduction, the reflectometer and the radiometer share the same RF-IF front-end, a commercial GPS down-converter. As a result both, radiometer and reflectometer, operate at 1575.42 MHz. However, it is known that for the best performance, radiometer should operate at the 1400-1427 MHz band and reflectometer at 1575.42 MHz. This non-optimal scenario is assumed because the aim of this prototype is to demonstrate the PAU concept and the scenario allows reducing the hardware resources required. On the other hand, we have to deal with some handicaps. The fist is the radiometer uses the 2.2 MHz GPS bandwidth, instead of the about 20 MHz available in L-band. Obviously, it has an impact on the radiometric sensitivity that can be compensated by increasing the integration time. The second handicap is the contamination that GPS signal can introduce in the radiometric measurements, which has to be quantified despite of the spread spectrum modulation GPS signal is lower than the noise level. Total GPS power density received over the Earth's surface is about -153 dBW/m^2^. The power received after a reflection over the Earth's surface depends on the receiver's height and on the antenna's effective area. In the worst case, where the specular reflection point is at the antenna's boresight and considering a surface reflection coefficient of Γ=0.7, a height of h=100 m and an antenna's effective area of A_eff_=0.16 m^2^ the radiometric measurement contribution is ΔT= P_r_/*k*_B_B=2.3 K (∼ 4.6 psu salinity error in warm water). However, if the reflection comes through the array pattern with an attenuation at least of 15 dB, the radiometric measurement contribution is just ΔT= 0.03 K (∼ 0.06 psu salinity error in warm water), below the 0.1 psu requirement of GODAE for open ocean [16]. Taking into account that the radiometer beam can be electronically steered, the chances of having a significant interference level are negligible.

PAU-RAD is a proof of concept of the PAU-SEOSAT/INGENIO [17] instrument that has been selected for the B phase of SEOSAT/INGENIO (stands for Spanish Earth Observation Satellite, Spanish contribution to GMES). satellite. In this PAU version the radiometer will operate in the 1404-1423 MHz band (bandwidth of 19 MHz) and the reflectometer at the L1 GPS frequency, using common RF chains and two separated IF chains.

### 2. 1. PAU-RAD analog receiver overview

**Figure 2.**
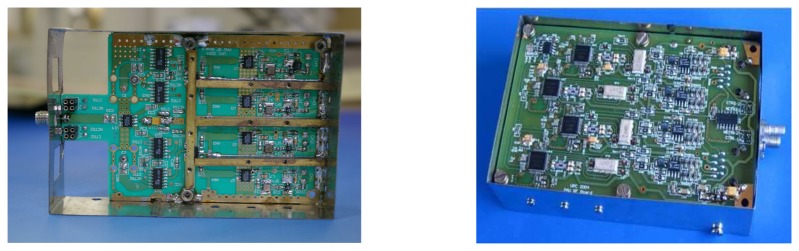
**a.** PAU's RF part of the elemental receiver with box. **b.** PAU's IF part of the elemental receiver with box.

Each receiver (Fig. 2) has two linear polarization antennas and two chains per polarization (four chains per receiver).

**Figure 3.**
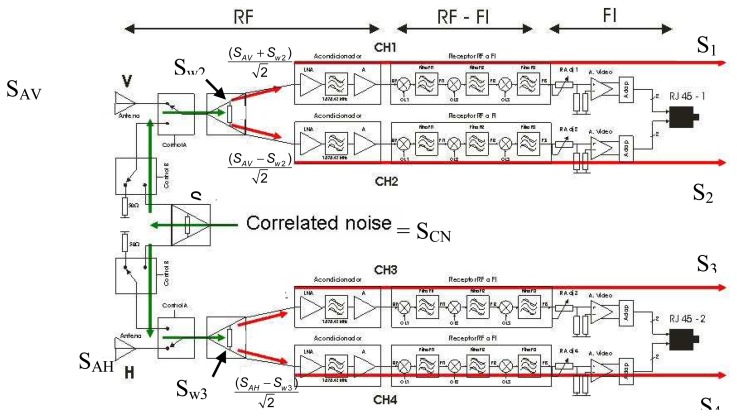
Analog receiver configuration for hardware calibration by noise injection.

A switch follows the antenna and commutes between the antenna signal (SA) and the correlated noise (SCN) (Fig. 3) from a controlled noise source (CNS), used for calibration purposes. A CNS has been designed to provide three temperature points (300, 400 and 500 K), so as to perform two types of calibration: internal hardware calibration and absolute radiometric calibration.

After the switch, the Wilkinson power splitter divides the signal in two, one for each receiver chain. The RF stage amplifies the signal by 33 dB. At the downconverter output the signals are centered at an IF of 4.309 MHz with a bandwidth of 2.2 MHz. The last stage is formed by the adjustable gain video amplifiers and an impedance matching network. From here the signals are sampled using 8 bits analog-digital converters (ADC). The fine amplitude adjustment is then performed in the digital part, using advanced processing algorithms as described in section 3.

### 2.2. Analog Acquisition

The number of bits used is a trade-off between the radiometric sensitivity of the full array, which depends on the clipping effect [18] and on the effective number of bits, and FPGA resources. The analysis developed on [19] shows that the best performance is given when the ADC's conversion window and the standard deviation of the signal is Vpp/σ_SIGNAL_ = 9.09, where V_pp_ means the window's voltage peak to peak. The same work analyzes the performance as a function of the number of bits, the result is that 8 bits is enough to neglect the digitization error and to ensure a main beam efficiency larger than 96 % of the digital beamformer.

Considering these results, the analog signals are sampled at 8 bits and 5.745 MHz. The sampling frequency is higher than the IF which allows a kind of “band-pass sampling” [20] downconverting the input signals at 0.25 times the digital frequency (1.436 MHz). Digitization of the 4 receiver chains (4 per element) is performed with 2 dual-channel 8-bit ADCs, which share the output bus. An internal multiplexer determines which digitalized channel is present at the ADC outputs. It is controlled by an 11.49 MHz signal, so in half-period of CLK each ADC is able to put its channels in the output bus. The control signals are all supplied by the FPGA to keep the synchronism with the internal digital processes.

Moreover, according to the relationship V_pp_= 9.09· σ_SIGNAL_ the signals amplitude have been adjusted to 110 mV_rms_ to clip less than 0.1% samples when the ADC dynamic range is set to 1 V. The digital output values go from 0 (-0.5V) to 255 (+ 0.5V), so a 128 offset value is present at the output data, that will have to be subtracted afterwards.

The signals from each element enter into the FPGA multiplexed in time by a factor of 4 (4 times faster). Since there are 16 elements, the processing unit must process 367.68 Mbytes of data per second. To process this data rate properly the hardware must operate, at least, at 4 times the sampling frequency (22.98 MHz).

### 2.3. PAU-RAD digital overview

The digital part of PAU-RAD has been split in a Control Unit (CU) that manages the system and another block called Arithmetic-Logic Unit (ALU), where the radiometer algorithms are implemented.

#### 2.3.1. PAU-RAD Arithmetic Logic Unit (ALU)

The PAU-RAD ALU processes the acquired data and finally computes the four Stokes parameters [21]. The digital implementation to compute the first two elements has been performed as follows:

(2a) $$I=\frac{1}{\tau_{i}}\int_{0}^{\tau_{i}}S_{1}\;(t)\cdot S_{2}^{*}\;(t)\;dt\to I=\frac{2}{\tau_{i}}\;(\;\langle I_{1}\cdot I_{2}\rangle +j\;\langle Q_{1}\cdot I_{2}\rangle )\;\text{and}$$

(2b) $$Q=\frac{1}{\tau_{i}}\int_{0}^{\tau_{i}}S_{3}\;(t)\cdot S_{4}^{*}\;(t)\;dt\to Q=\frac{2}{\tau_{i}}\;(\langle I_{3}\cdot I_{4}\rangle +j\;\langle Q_{3}\cdot I_{4}\rangle )\;,$$

where:

- *S*_1_ and *S*_2_ are the signals from the vertical antenna with the Wilkinson noise (Wilkinson noise is defined as the noise generated in the Wilkinson load) and the receiver noise, (*S*_1,2_ signals are deeply analyzed on 3.4),
- *S*_3_ and *S*_4_ are the signals from the horizontal antenna with the Wilkinson noise and the receiver noise, (*S*_3,4_ signals are deeply analyzed on 3.4),
- *I*_1,2,3,4_ and *Q*_1,2,3,4_ are the in-phase and quadrature signals for *S*_1,2,3,4_ signals, respectively, and
- *τ_i_* is the integration time.

And the digital implementation to compute the second two elements has been performed as follows:

(2c) $$U=\frac{1}{\tau_{i}}\int_{0}^{\tau_{i}}S_{1}\;(t)\cdot S_{3}^{*}\;(t)\;dt\to U=\frac{2}{\tau_{i}}\;(\langle I_{1}\cdot I_{3}\rangle )\;\text{and}$$

(2d) $$V=\frac{1}{\tau_{i}}\int_{0}^{\tau_{i}}S_{1}\;(t)\cdot S_{3}^{*}\;(t)\;dt\to V=\frac{2}{\tau_{i}}\;(\langle Q_{1}\cdot I_{3}\rangle )\;,$$

where:

- *S*_1_ and *S*_2_ are the signals from the vertical antenna with the Wilkinson noise and the receiver noise,
- *S*_3_ and *S*_4_ are the signals from the horizontal antenna with the Wilkinson noise and the receiver noise,
- *I*_1,3_ and *Q*_1,3_ are the in-phase and quadrature signals for *S_1,3_* signals, respectively, and
- *τ_i_* is the integration time.

In order to save FPGA resources and maximize their use, signal time-multiplexing and hardware reuse techniques have been intensively used. This increases the synchronism complexity because it is necessary to relate the ADC output to a synchronous signal to know which signals are being processed at each clock cycle. Furthermore, to improve the maximum operating frequency two techniques are used:

- parallelization, that involves the use of sequential circuits (changes a multiple cycle serial operation to a multiple stage parallel operation), and
- pipelining, that involves the use of combinational circuits (changes a high delay combinational circuit to multiple low delay circuits with registers).

In these conditions the digital design operating frequency must be the same as the sampling frequency. Actually, data is processed at twice the sampling frequency in order to obtain a second steered beam.

**Figure 4.**
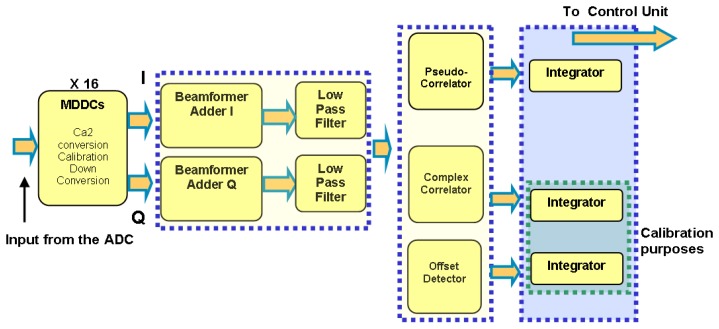
PAU-ALU-RAD diagram.

The data path is schematically shown in Fig. 4. A correction must be first applied to the input signals to adjust their level before being processed. This is related to the output of the ADC that gives a binary quantification of the analog signal between 0 and 255. It is therefore necessary to subtract 128 to all the signals while, at the same time, converts the signals into 2's complement.

In order to perform the beamforming and the pseudo-correlation, all channels must have a zero relative phase and the same amplitude. Phase errors are largely related to the fact that the 64 analog receivers do not share the same LO. A 10 MHz clock reference is distributed to all receivers' PLLs, which lock with a random phase what is uniformly distributed within 0 to 2π radians. Amplitude errors are introduced by gain variations (aging and drifts) from each receiver's chain, and are adjusted within ±1 dB.

As discussed in section 2.2, the incoming signals are centered at 0.25 times the digital frequency. Then, it is possible to simultaneously down-convert, apply the beam steering phase, and perform the phase and amplitude calibration of the signals with a single multiplication between the signal and the digital local oscillator signal:

(3) $$S_{LO}\;(n)=\alpha \exp(-j\;(w_{n}n-\theta_{\mathrm{beam}}))\;\text{with}\;n=1,2,3,\ldots$$

where:

- *w_n_*= 0.25 times the digital frequency,
- *α*: complex calibration coefficient (amplitude and phase) and
- *θ_beam_*: beam steering phase.

**Figure 5.**
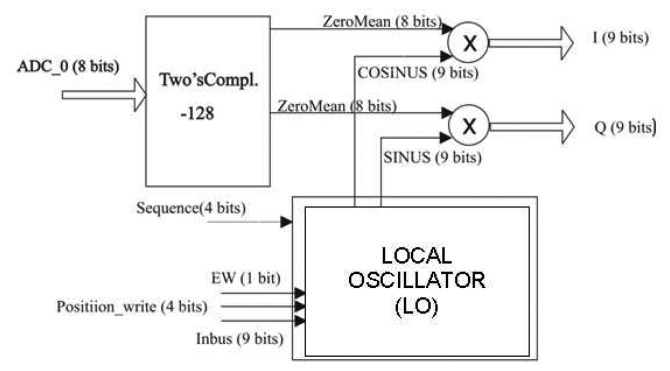
MDDC sketch.

To synthesize this in the digital domain, a Multiplexed Digital Down-Converter (MDDC) block has been developed (Fig. 5) which is also fully adaptive to create new beams or dynamically compensate the phase/amplitude error correction. The MDDC consists of a phase and amplitude variable Local Oscillator (LO), whose values are determined by the PAU-RAD control unit (CU) as a function of the beam steering, and the estimated phase and amplitude errors. The LO is updated when a new beam is synthesized or the instrument recalibrated. Note that there are 16 MDDCs and 64 LOs, one for each receiver.

After the MDDC the digital beamformer signal is formed by adding the 16 signals that came from the receivers using a weighted adder. The next step is to low-pass filter the I/Q composed beams signals, to do this an IIR filter has been chosen instead of FIR one, due to the smaller order required to obtain the same attenuation. Once the signals are filtered, they are input to the pseudo-correlator block that multiplies them by pairs (Eqn. 2), the results are accumulated in the integrator block.

PAU-RAD CU switches the input analog receiver signal from antenna signal to a correlated noise signal to estimate the phase and amplitude errors. The correlated noise is needed to determine the relative amplitude and phase errors between channels and calibrate them.

#### 2.3.2. PAU-RAD Control Unit (CU)

PAU-RAD CU is built on an embedded processor which is implemented in the FPGA. It is a 32 bit processor with 16 bit instructions specially developed to be programmed in C language. PAU-RAD CU has three main functions:

- it is both, the interface with the PAU-RAD ALU and with the external interface of PAU-RAD; controlling the system operation mode and transmitting to PAU-RAD ALU the state,
- computes the corrections to be applied at the digital LO to calibrate the signals, and
- attends the remote control based on a TCP/IP connection.

**Figure 6.**
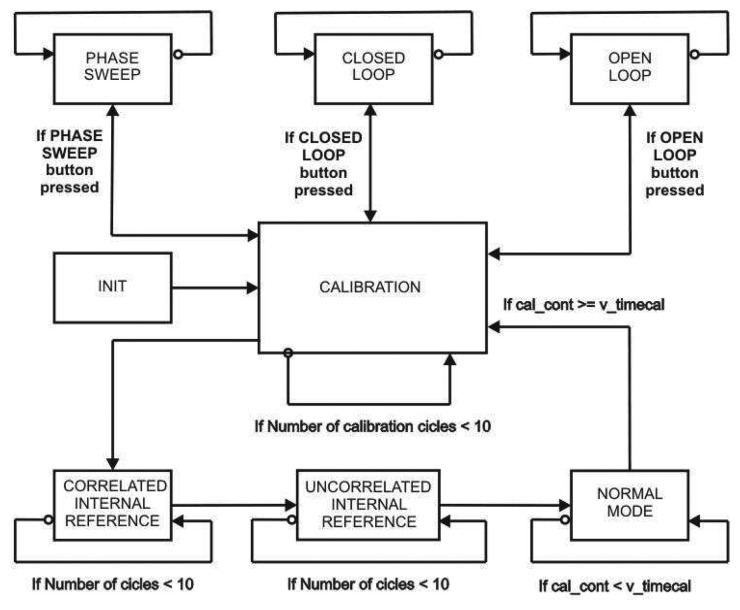
PAU-RAD CU extended datagram.

PAU-RAD CU has been designed as a finite state machine, which is always working in one of the different states according to the radiometer's operation mode at each time instant. The block diagram in Fig. 6 shows the different states of PAU-RAD CU and the transitions between them. There is a C main function to implement this State Diagram, and a subroutine to each state, therefore the descriptions of the different states are an explanation of the corresponding subroutine features. There are two types of states: the extended ones that perform test functionalities (PHASE SWEEP (PS), CLOSE LOOP (CL) and OPEN LOOP (OL)), and the fundamental ones that perform the core of the radiometer functions (INITIALIZATION (INIT), CALIBRATION (CAL), CORRELATED INTERNAL REFERENCE (CIR), UNCORRELATED INTERNAL REFERENCE (UIR) and ANTENNA MEASUREMENT (AM)).

The different states are described below:

- INIT: initialization state. It has two main functions:
  - sets the default parameters of the system when resetting and
  - it listens the remote commands coming from the external interface. In a reset process the PAU-RAD CU transmits to the PAU-RAD ALU the system timer.
- CAL: hardware calibration state. It switches the input signal to the correlated noise source (CNS) and starts the phase and amplitude error estimation. The LO is modified using the correction values once the error is achieved.
- CIR: internal radiometric calibration state. It switches the input signal to two well known temperature points of the CNS. The signals are correlated and sent to the external interface through the TCP/IP connection. These results are used to set a unique relationship between the detected power and the brightness temperature. The aim of this state is to reduce the time between external hot-cold calibrations.
- UIR: offset estimation state. It switches the input signal of each chain to a matched load and correlates the signals. The result is the offset correction to be applied at the radiometric measures.
- AM: Computes the Stokes parameters. It switches the input signal to the antennas. It is the state in which the system spends most of the time.
- OL: temporal phase and amplitude drift analysis state, only used for test purposes. It switches the input signal to a CNS' predefined point. LOs are set to the same amplitude and phase, them the power and the phase of each chain is computed.
- CL: phase and amplitude calibration performance state, used only for test purposes. It switches the input signal to a CNS' predefined point. The system is always estimating and correcting the phase and amplitude errors.
- PS: LO phase sweep analysis state, only used for test purposes. It switches the input signal to a CNS' predefined temperature point. The system changes constantly the LO's phase and computes the correlation between channels.

The standard sequence (CAL-CIR-UIR-AM) is only modified if a remote command has been received. In this case, the system moves to INIT state. The time spent in each state depends on the hardware stability. The variable v_timecal is a timer that can be configured depending on the scenario and determines the inter-calibration period. It is the time that the system spends in the AM. Each fundamental cycles, except AM, are executed some number of times before the next state.

Finally, the CU has a remote reset watchdog (the reset signal comes from TCP/IP connection) to periodically check the status of the connection, and reset the CU if necessary.

## 3. Calibration algorithms implementation and tests

In order to test and improve the calibration algorithms for PAU a one-receiver version has been developed (PAU-OR) [22]. It includes PAU-RAD and PAU-GNSS/R, but not PAU-IR.

**Figure 7.**
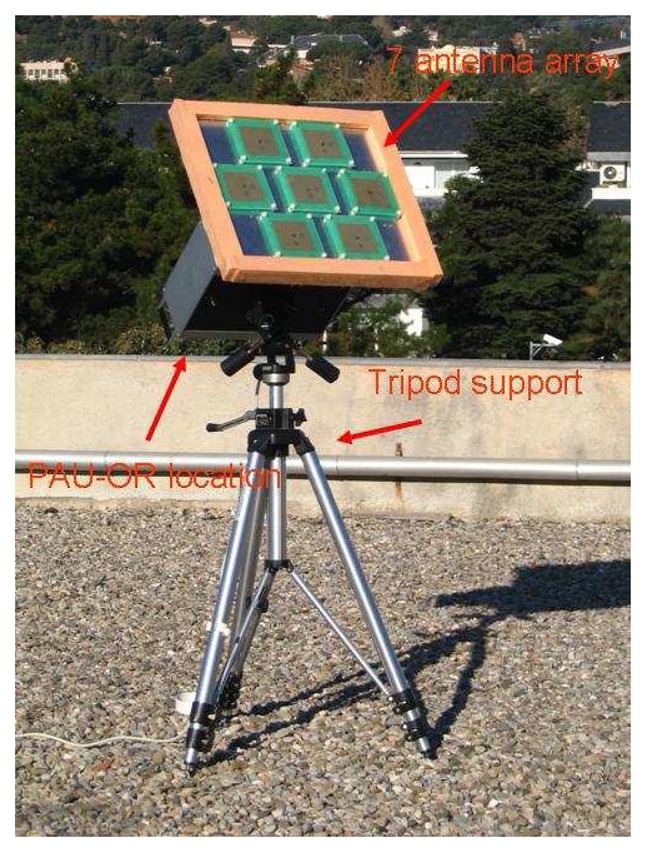
PAU-OR general view.

PAU-OR (Fig. 7) has a full polarization seven-patch array antenna with 25° beamwidth and 17.5 dBi directivity. Analog circuitry is located inside a thermal isolated and regulated box using Peltier cells (achieving a thermal stability, standard deviation of the internal temperature, lower than 0.3°C). A left hand circularly polarized signal is composed and it is sent to PAU-GNSS/R. A 15 dB ENR noise source is used for the CNS to perform the hardware calibration and for internal reference radiometric calibration.

In PAU-OR, and by extension in PAU-RAD, there are two types of calibration. The first one is related to the internal relative calibration between the analog receivers that have different gains and phases. Needed to perform the beamforming and operates as a pseudo-correlation radiometer. The second one is the radiometric calibration to set a unique relationship between the cross-correlation outputs and the Stokes elements.

The CNS has been designed to achieve these two types of calibration, allowing the generation of three different temperature points.

### 3.1. Internal relative phase and amplitude calibration

The analog receiver (Fig. 3) has two possible inputs: one from the antenna (MA mode) and the other from the CNS, for calibration purposes (CIR mode). By commuting the input switch to the correlated noise input the same signal is applied at the input of each chain, which allows estimating the amplitude and phase differences among them. The equations obtained from PAU are extensively developed in [12] where all non-ideal terms such as the Wilkinson power splitter unbalance or the imperfect matching and isolation are taken into account. Note that in this paper the system is assumed to be ideal to simplify the mathematics and to clarify the calibration algorithm. If the system imperfections are accounted for, additional terms appear, but cancel out in the differential algorithm described below.

When the input of the analog receivers is commuted to the CNS path, the four ideal output signals are given by:

(4a) $$S_{1}=\frac{1}{\sqrt{2}}\left(\frac{1}{\sqrt{2}}(S_{CN}+S_{W1})+S_{W2}\right)+S_{\text{REC}1},$$

(4b) $$S_{2}=\alpha \;(\frac{1}{\sqrt{2}}\left(\frac{1}{\sqrt{2}}(S_{CN}+S_{W1})-S_{W2}\right)+S_{\text{REC}2})\;,$$

(4c) $$S_{3}=\beta \;(\frac{1}{\sqrt{2}}\left(\frac{1}{\sqrt{2}}(S_{CN}-S_{W1})+S_{W3}\right)+S_{\text{REC}3})\;\text{and}$$

(4d) $$S_{4}=\gamma \;(\frac{1}{\sqrt{2}}\left(\frac{1}{\sqrt{2}}(S_{CN}-S_{W1})-S_{W3}\right)+S_{\text{REC}4})\;,$$

where signals are defined as effective voltage waves:

- S_CN_ : is the noise signal from the CNS (same for the four channels),
- S_w1_: is the thermal noise signal generated at the first Wilkinson power splitter that divides the noise from the CNS in two, one for the upper branch (chains 1 and 2, Fig. 3) and the other for the lower branch (chains 3 and 4, Fig. 3),
- S_w2_ and S_w3_: are the thermal noise signals from the second and third Wilkinson power splitter, at the upper branch and at the lower branch respectively. Note that these signals are uncorrelated among them,
- S_REC1_, S_REC2_, S_REC3_ and S_REC4_: are the thermal noise signals introduced by from each receiver. Note that these signals are also uncorrelated among them, and
- *α*, *β*, *γ*: are the complex coefficients that contain the relative phase and amplitude error with respect to the first channel.

To estimate the complex coefficients *α*, *β*, *γ*, the following correlations are obtained using the signals defined in Eqn. 4:

(5a) $$<S_{1}\cdot S_{1}^{*}>=\frac{T_{CN}+T_{W1}}{4}+\frac{T_{W2}}{2}+T_{\text{REC}1}\;,$$

(5b) $$<S_{1}\cdot S_{2}^{*}>=\alpha \;(\frac{T_{CN}+T_{W1}}{4}-\frac{T_{W2}}{2})\;,$$

(5c) $$<S_{1}\cdot S_{3}^{*}>=\beta \;(\frac{T_{CN}-T_{W1}}{4})\;\text{and}$$

(5d) $$<S_{1}\cdot S_{4}^{*}>=\gamma \;(\frac{T_{CN}-T_{W1}}{4})\;,$$

where:

- T_CN_: noise temperature from the CNS, since *T_CN_* □ 〈|*S_CN_*|^2^〉 (Note that the equivalence to brightness temperature has been normalized by kB;.
- T_w1_: noise temperature introduced by first Wilkinson power splitter, note that *T_W_*_1_ □ *T_ph_*_1_ □ *T_ph_*_2_, that are the receivers physical temperature and
- T_w2_: noise temperature introduced by the upper branch Wilkinson power splitter, note that *T_W_*_2_ □ *T_ph_*_2_ □ *T_ph_*_1_, that are the receivers physical temperature.

In order to compare Eqns. 5a and 5b with 5c and 5d the $\pm \overset{T_{W2}}{\;}/\underset{2}{\;}$ and *T_REC_*_1_ terms that must be cancelled. To eliminate these terms a differential calibration approach is used, by injecting two different noise temperatures (T_CN1_ and T_CN2_), and subtracting the results for each channel:

(6a) $$<S_{1}\cdot S_{1}^{*}{>}_{T_{CN1}}-<S_{1}\cdot S_{1}^{*}{>}_{T_{CN2}}=\frac{T_{CN1}-T_{CN2}}{4}\;,$$

(6b) $$<S_{1}\cdot S_{2}^{*}{>}_{T_{CN1}}-<S_{1}\cdot S_{2}^{*}{>}_{T_{CN2}}=\alpha \;(\frac{T_{CN1}-T_{CN2}}{4})\;,$$

(6c) $$<S_{1}\cdot S_{3}^{*}{>}_{T_{CN1}}-<S_{1}\cdot S_{3}^{*}{>}_{T_{CN2}}=\beta \;(\frac{T_{CN1}-T_{CN2}}{4})\;\text{and}$$

(6d) $$<S_{1}\cdot S_{4}^{*}{>}_{T_{CN1}}-<S_{1}\cdot S_{4}^{*}{>}_{T_{CN2}}=\gamma \;(\frac{T_{CN1}-T_{CN2}}{4})\;,$$

The complex coefficients α, β and γ are them obtained without the “a priori” knowledge of the receiver's noise temperature. The digital LO is then modified and, as a result, the channels are calibrated.

Note that:

- Equation 5 and Eqn. 6 the kB terms have been deliberately omitted so that “powers” are directly expressed as temperatures (Kelvin). This analysis can be directly extended to the case of non-ideal power splitters and receivers as detailed in [12], and
- As is said, the previous analysis has assumed that the Wilkinson power splitters were ideal, and that all inputs were perfectly matched. A detailed analysis was performed in [12] which shows that when the differential technique is used, the non-ideal terms are cancelled out, thus reducing to Eqn. 6.

### 3.2. Test of the internal relative phase and amplitude calibration algorithms

**Figure 8.**
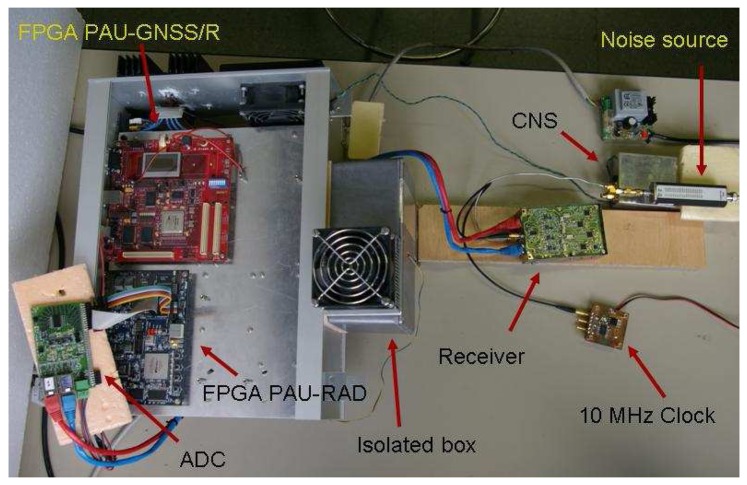
PAU-OR general view, set up for test purposes.

A set of test has been performed in order to evaluate the algorithms performance. All these tests have been done in CL mode, the system is always injecting two noise levels to estimate and correct the phase and amplitude errors on a snap-shot basis every 0.53 s. (integration time). Figure 8 shows the test set-up. Note that the receiver is outside the thermal isolated box to show the whole test set-up.

**Figure 9.**
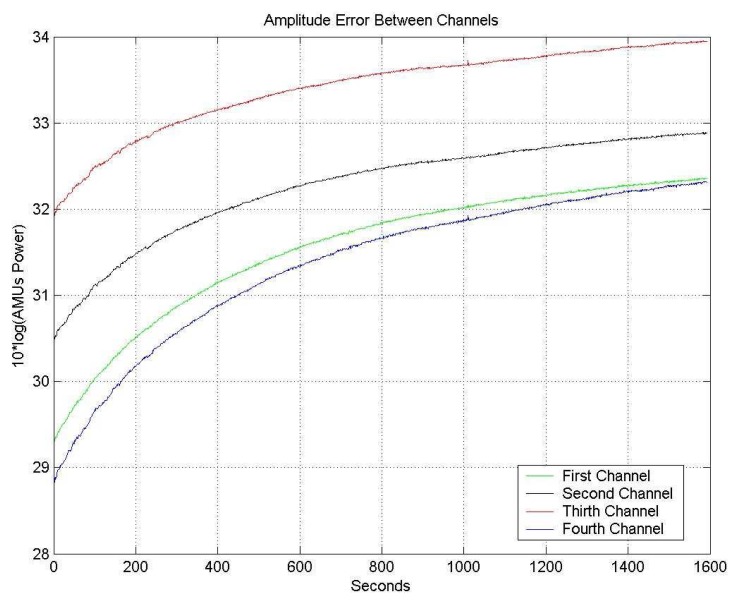
One receiver amplitude temporal drifts, without any correction.

Figure 9 shows the different output power (in counts: arbitrary measurement units, AMU), during the warm up transient, for the four receiving channels. After a warm-up transient of 50 min., the amplitude values stabilize, and the calibration algorithm can then be applied.

**Figure 10.**
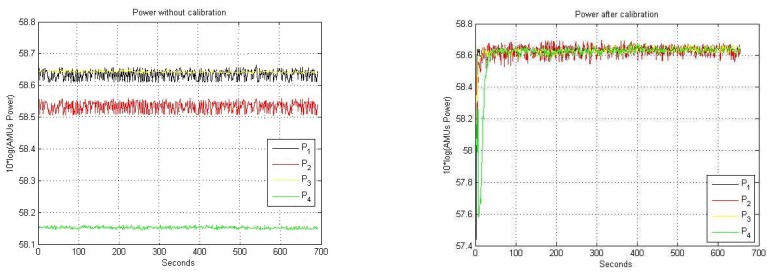
**a.** One receiver amplitude temporal drifts, without any correction. **b.** One receiver amplitude after the differential amplitude calibration algorithm.

After applying the calibration algorithm at each measurement. The means and the standard deviations for each channel are m1,2,3,4= 58.63 dBAMU, within the goal of ±0.1 dB residual amplitude calibration error, and σ_1_= 0.017, σ_2_= 0.032 σ_3_= 0.016 and σ_4_= 0.015 dBAMU, respectively. (Fig. 10b)

Note that in Fig. 10a the powers are expressed in arbitrary measurement units (AMU, 1 AMU = 1 count). Before calibration the mean and the standard deviation for each channel are m_1_= 58.63, m_2_= 58.53, m_3_= 58.64 and m_4_= 58.15 dBAMU and σ_1_= 0.015, σ_2_= 0.016 σ_3_= 0.003 and σ_4_= 0.003 dBAMU, respectively.

**Figure 11.**
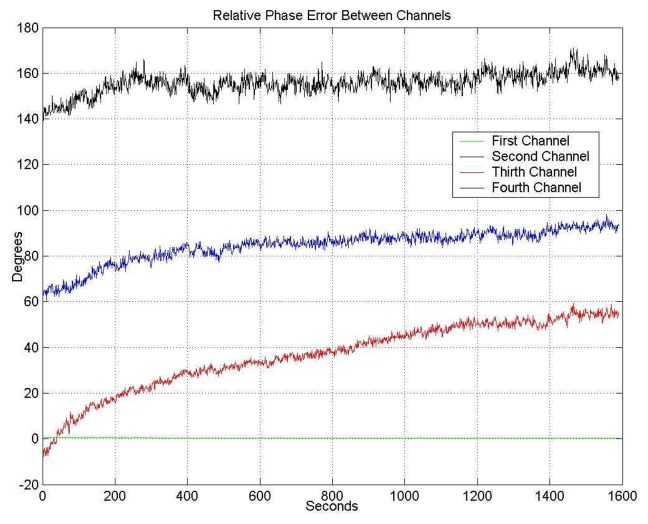
Phase temporal drifts of the four channels, without any correction.

Figure 11 shows the phase drift of the four channels just after turning on the receiver. Again, after a warm-up transient of 50 min., the phase values stabilize, and the calibration algorithm can be applied.

**Figure 12.**
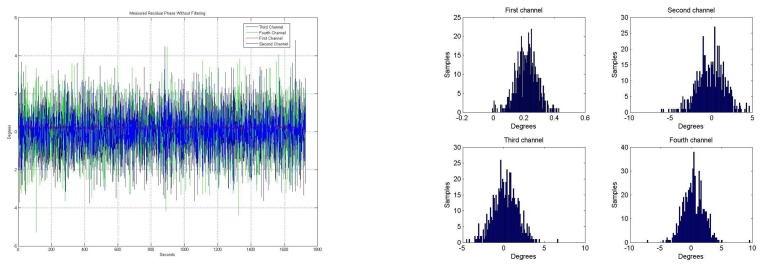
**a.** Residual phase error, after the phase calibration. **b.** Residual phase error histogram after the phase calibration.

Figure 12 shows the residual phase error as the result of the application of the described algorithm applied. After the transient period, the residual phase error standard deviations are: σ_1_= 0.069°, σ_2_= 1.343°, σ_3_= 1.116° and σ_4_= 1.302°.

**Figure 13.**
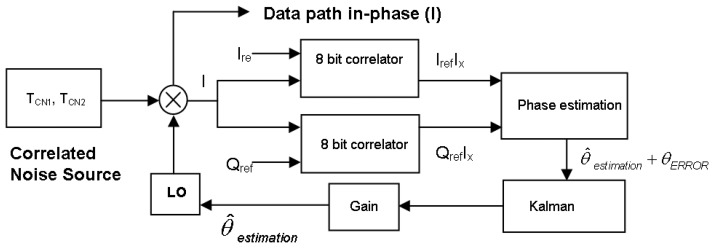
Digital hardware calibration for phase error estimation using a Kalman filter.

In order to reduce even further the residual phase error a closed-loop Kalman filter [15] has been implemented (Fig. 13). A Kalman filter is an efficient recursive filter that estimates the state of a dynamic system from a series of incomplete and noisy measurements, in this case the filter's input is the estimated phase error that contains measurement noise. The filter's output is the estimated phase error with smaller noise level.

There are two main parameters in a Kalman filter to be tuned: the standard deviation of the noisy signal and the gain coefficient. The first one, the measurement of the standard deviation of the noisy signal, was experimentally determined through several iterations. The second one, the filter gain, is a trade-off between the convergence speed and the residual error and the best results have been obtained with a 0.95 gain.

**Figure 14.**
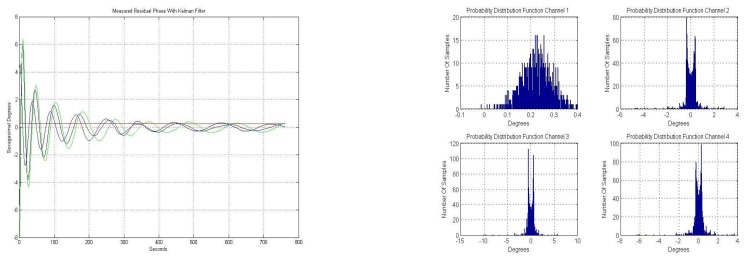
**a.** Residual phase error, using a closed-loop Kalman filter. **b.** Phase error residual histogram, using a closed-loop Kalman filter.

Figure 14a shows the temporal evolution of the residual phase error using a closed-loop Kalman filter. After a transient of about 100 s., when there is some ringing, the residual phase error stabilizes to smaller values.

Figure 14b shows the phase error histograms of the residual phase error using a closed-loop Kalman filter. The standard deviations of the phase of each channel are: σ_1_= 0.012°, σ_2_= 0.227°, σ_3_= 0.278° and σ_4_= 0.189°, while the means of each of the four channels is zero. These residual standard deviation values are very low and allow performing the pseudo-correlation radiometer and the digital beam-forming accurately.

Note that the histograms (Fig. 14b) look like those of a sinusoidal histogram due to the sinusoidal oscillations of the Kalman transient ringing.

### 3.3. PAU hardware calibration global algorithm

This algorithm is implemented in the PAU-RAD-UC, the algorithm has six steps that can be summarized as follows:

- Switching the input to the CNS input, using the highest temperature point available, and acquisition of one set of data from the PAU-RAD-ALU.
- Data acquisition from the PAU-RAD-ALU in order to estimate the relative phase error. For each channel compute and set the new error phase values for the LO.
- Data acquisition from the PAU-RAD-ALU in order to estimate the amplitude error, first acquisition.
- Modification of the CNS to obtain the medium noise temperature point and acquire data from the PAU-RAD-ALU in order to estimate the amplitude error, second acquisition.
- Compute and set the final values for the LO, taking into account phase and amplitude errors, and steering phase.

### 3.4. Radiometric absolute calibration using internal references

The classical method to calibrate a radiometer consists of using (at least) an external T_HOT_, (eg. microwave absorber) and an external T_COLD_, (eg. the clear sky). Using these well-known reference points, an unique relationship between the antenna temperature and the measured power is obtained. This method requires a mechanical movement of the antenna. To avoid this inconvenience, a radiometric calibration based on internal temperature references has been implemented. The CNS system generates a third well-known temperature point at the input of the receiver chain. The input noise is the same as that of a microwave absorber placed in front of the antenna, both at the same physical temperature than the matched load. With these results and using a previous measurement of T_SKY_ (which obviously includes the antenna Ohmic losses, η_Ω_) the correction coefficients can be obtained.

## 4. Conclusions and future work

The calibration algorithm of PAU-RAD has been presented on a one-receiver demonstrator: PAU-OR. The instrument has been implemented, the calibration algorithms tested and the result presented and discussed. On one hand, to take advantage of using the same band for both reflectometer and the radiometer, its beam must be steered to a direction where the GNSS-reflected signal is attenuated by at least 15 dB (ΔT= 0.03 K). On the other hand, the reflectometer beam must be pointed to the specular reflection.

The digital design can be easily modified to generate two simultaneous beams in real-time. The whole design, analog and digital parts, has been satisfactorily tested. Moreover, the PAU-RAD CU has been designed in C language over a microprocessor giving it versatility. The state diagram has been developed to obtain the best performance in any kind of scenario and allows changing the main radiometer's parameters.

The main results are summarized below:

- Differential amplitude and phase calibration algorithms have been developed in order to mitigate the offset introduced by the subsystems non-idealities and to give robustness to the error estimation.
- The results obtained with de differential amplitude calibration algorithm are better than the required 0.1dB: σ_1_= 0.017, σ_2_= 0.032 σ_3_= 0.016 and σ_4_= 0.015 dBAMU.
- The results obtained with the differential phase calibration algorithm are smaller than 1.5°: σ_1_= 0.069°, σ_2_= 1.343°, σ_3_= 1.116° and σ_4_= 1.302°, and
- the improvement over the standard phase calibration algorithm filtering using a Kalman filter improves the results obtained, with standard deviations smaller than 0.5°: σ_1_= 0.012°, σ_2_= 0.227°, σ_3_= 0.278° and σ_4_= 0.189°.

Future work will focus on exporting the know-how and the techniques developed in PAU-OR to the 16 element PAU.
